# The Role of NLRP3 in Regulation of Antimicrobial Peptides and Estrogen Signaling in UPEC-Infected Bladder Epithelial Cells

**DOI:** 10.3390/cells12182298

**Published:** 2023-09-18

**Authors:** Anna Lindblad, Rongrong Wu, Katarina Persson, Isak Demirel

**Affiliations:** School of Medical Sciences, Örebro University, 701 82 Örebro, Sweden; anna.lindblad@oru.se (A.L.); rongrong.wu@oru.se (R.W.); katarina.persson@oru.se (K.P.)

**Keywords:** NLRP3 inflammasome, estradiol, antimicrobial peptides, uropathogenic *Escherichia coli*, urinary tract infections

## Abstract

The NLRP3 inflammasome, estrogen and antimicrobial peptides have all been found to have a vital role in the protection of the bladder urothelium. However, the interdependence between these protective factors during a bladder infection is currently unknown. Our aim was to investigate the role of NLRP3 in the regulation of antimicrobial peptides and estrogen signaling in bladder epithelial cells during a UPEC infection. Human bladder epithelial cells and CRISPR/Cas9-generated NLRP3-deficient cells were stimulated with the UPEC strain CFT073 and estradiol. The gene and protein expression were evaluated with microarray, qRT-PCR, western blot and ELISA. Microarray results showed that the expression of most antimicrobial peptides was reduced in CFT073-infected NLRP3-deficient cells compared to Cas9 control cells. Conditioned medium from NLRP3-deficient cells also lost the ability to suppress CFT073 growth. Moreover, NLRP3-deficient cells had lower basal release of Beta-defensin-1, Beta-defensin-2 and RNase7. The ability of estradiol to induce an increased expression of antimicrobial peptides was also abrogated in NLRP3-deficient cells. The decreased antimicrobial peptide expression might be linked to the observed reduced expression and activity of estradiol receptor beta in NLRP3-deficient cells. This study suggests that NLRP3 may regulate the release and expression of antimicrobial peptides and affect estrogen signaling in bladder epithelial cells.

## 1. Introduction

Urinary tract infection (UTI) is a common infection in humans, particularly in women. Approximately 50% of all women will suffer from at least one UTI during their lifetime, and 25% of the women experiencing a first UTI will have an increased risk of recurrency within 6 months [1,2]. Most UTIs are caused by uropathogenic *Escherichia coli* (UPEC), which expresses a variety of virulence factors, such as capsule, lipopolysaccharides (LPS), type-1 fimbriae, α-hemolysin (HlyA), P-fimbriae, Toll/interleukin-1 receptor domain-containing protein C (TcpC), sisA, sisB and iron acquisition systems to successfully colonize the bladder [3,4,5,6]. The attachment of UPEC to host receptors on the superficial bladder epithelial cells is mediated by type-1 fimbriae. Following attachment, UPEC invades the cells [7] and establishes intracellular bacterial communities (IBCs) [7,8]. UPEC can also invade deeper layers of the urothelium and form quiescent intracellular reservoirs where it can persist for months and give rise to recurrent infections [9,10].

Inflammasomes are multiprotein complexes that can evoke an immune response by the activation of caspase-1 and the subsequent maturation and release of IL-1β and IL-18 [11]. The most-studied inflammasome is the nucleotide oligomerization domain (NOD)-like receptor pyrin domain-containing 3 (NLRP3) inflammasome. The assembly and activation of the NLRP3 inflammasome is regarded as a two-step process during canonical activation. It is initiated when pattern recognition receptors (PRRs), such as toll-like receptors (TLRs), sense pathogen-associated molecular patterns (PAMPs) or nucleotide oligomerization domain (NOD)-like receptors (NLRs) sense danger-associated molecular patterns (DAMPs). The activation of NLRP3 leads to the maturation of IL-1β and IL-18 by caspase-1. Caspase-1 activation leads to the cleavage of gasdermin D (GSDMD), which initiates pyroptosis and the release of IL-1β and IL-18 [11,12]. Recent studies have shown that NLRP3 has an important role in the inflammatory response during a UTI caused by UPEC [13,14,15,16,17,18,19]. Interestingly, UPEC strains expressing TcpC can inhibit formation of the NLRP3 complex, which disrupts the host immune system [20]. We have previously showed that NLRP3 is involved in the host–pathogen interaction during a UTI by altering the cytokine and chemokine release from bladder epithelial cells and by altering the antimicrobial activity of neutrophils [21].

Antimicrobial peptides (AMPs), part of the innate immune system, are small polypeptides (<10 kDa) that have direct antimicrobial activity or immunomodulatory functions [22]. The expression of antimicrobial peptides in the urothelial tract is well-studied, and they contribute to maintaining a healthy microflora. They are produced by the urothelium of the bladder, urethra, ureter and kidneys. In the urinary tract, the most common AMPs are cathelicidin, human β-defensins, human α-defensin 5 (HD5) and RNase7 [22]. However, the association between NLRP3 and its involvement in AMP production in the urinary tract is unknown. Current evidence indicates that the production of AMPs is regulated by the sex hormone estrogen, which is known to support many different aspects of the urothelial defense mechanism [23]. There are two types of nuclear estrogen receptors (ER), ER alpha and ER beta, and they have different functions and tissue expression patterns. ER beta is the major ER type in the urinary bladder [24,25], and it is involved in epithelial differentiation and maintenance [25,26]. ER alpha is more often expressed in the vagina [25,26]. The circulating level of estrogen varies during the menstrual cycle, which influences the prevalence of symptoms from the urinary tract. NLRP3 is known to be important for the host defense of the urinary tract against UPEC, but the knowledge of how NLRP3 contributes to host defense mechanisms involving estrogen in bladder epithelial cells is limited. The aim of this study was to investigate the role of NLRP3 in the regulation of AMPs and estrogen signaling in bladder epithelial cells during an experimental UPEC infection.

## 2. Material and Methods

### 2.1. Human Bladder Epithelial Cells

The 5637-cell line (ATTCC HBT-9), purchased from the American Type Culture Collection (Manassas, VA, USA), was cultured in Dulbecco’s modified Eagle Medium (DMEM) (Thermo Fisher Scientific, Walthman, MA, USA) and complemented with 1 mM non-essential amino acids, 10% fetal bovine serum (FBS) and 2 mM L-glutamine (Thermo Fisher Scientific, Walthman, MA, USA) at 37 °C with 5% CO_2_. During infection experiments, DMEM containing 2% FBS, 1 mM non-essential amino acids and 2 mM L-glutamine was used, as previously described [27]. During estradiol experiments, cells were serum-starved overnight. DMEM containing 2% charcoal stripped FBS, 1 mM non-essential amino acids and 2 mM L-glutamine was then used.

### 2.2. CRISPR/Cas9 Genome Editing

The pSpCas9 (BB)-2A-Puro (PX459, V2.0) plasmid (Addgene plasmid # 62988) [28] was used for CRISPR/Cas9 gene editing in 5637 cells. Plasmid transfection was conducted using Lipofectamine 2000 (Life Technologies, Carslbad, CA, USA). The target site was GCTAATGATCGACTTCAATG (NLRP3). Puromycin (2.5 µg/mL, Sigma-Aldrich, St. Louis, MO, USA) selection was carried out 24 h after transfection. All experiments were performed utilizing a polyclonal mixture of gene-edited bladder epithelial cells. Verification of the phenotype was carried out through Western Blot analysis, as previously outlined [19].

### 2.3. Bacterial Strains and Growth Conditions

The UPEC strain CFT073 was used for all infection experiments. This strain was isolated from an individual with acute pyelonephritis [29]. Prior to infection experiments, CFT073 was grown in Difco Luria-Bertani (LB) broth (Lennox; Franklin Lakes, NJ, USA) aerobically at 37 °C on a shaker overnight, as previously described [27].

### 2.4. Microarray Analysis

The 5637-bladder epithelial cell line (Cas9 controls and NLRP3-deficient cells) was infected with CFT073 for 6 h at a multiplicity of infection (MOI) of 10 at 37 °C with 5% CO_2_. Total RNA was isolated from the cells utilizing the E.Z.N.A. Total RNA Kit I (Omega Bio-tek, Norcross, GA, USA). RNA quality and integrity was assessed using the Agilent Tapestation 2200 platform (Agilent Technologies, Palo Alto, CA, USA). The RNA integrity number (RIN) was 10 for all samples. The Low Input Quick Amp WT Labelling Kit (Agilent) was used to label cRNA. Hybridization of labelled cRNA samples was carried out in a G2545A hybridization oven (Agilent) onto Agilent SurePrint G3 (v3) Human Gene Expression 8 × 60 k (Agilent Technologies, Palo Alto, CA, USA) glass arrays. The arrays were then scanned with a G2505C array laser scanner (Agilent Technologies, Palo Alto, CA, USA). The feature Extraction Software (v. 10.7.3.1, Agilent Technologies, Palo Alto, CA, USA) was used for image analysis and data extraction [30]. Gene expression data is available in the GEO database with the accession number GSE243098.

### 2.5. Stimulation of Bladder Epithelial Cells and Conditioned Medium

The 5637-bladder epithelial cell line (Cas9 controls and NLRP3-deficient cells) was infected with CFT073 for 6 h at MOI 10 and incubated at 37 °C with 5% CO_2_. Supernatants were centrifuged at 5000 g for 5 min and sterile filtered to remove the bacteria before freezing the samples at −80 °C, as previously described [21]. These supernatants were defined as conditioned medium. Plating on TSA agar was conducted to ensure that no viable bacteria were found. Bladder epithelial cells were also stimulated with 1 nM or 10 nM estradiol for 6 or 24 h at 37 °C with 5% CO_2_. DMSO was used as a negative control.

### 2.6. Measurement of Antimicrobial Peptides

ELISA was performed to measure Beta-defensin 1 (NBP2-67933, Bio-techne, Minneapolis, MN, USA), Beta-defensin 2 (NBP2-77363, Bio-techne), LL37 (NBP3-06932, Bio-techne, Minneapolis, MN, USA) and RNase7 (ab215418, Abcam, Cambridge, UK), according to the manufacturer’s instructions.

### 2.7. Bacterial Growth Assay

Conditioned medium from Cas9 controls and NLRP3-deficient cells were transferred to a 96-well plate and stimulated with 1 × 10^6^ CFU/mL CFT073. The plate was then incubated at 37 °C and the optical density (600 nm) was measured every hour using a spectrophotometer (Cytation 3, Biotek Inc., Winooski, VT, USA).

### 2.8. RNA Isolation and Real Time RT-PCR

Total RNA from 5637 cells was isolated with the E.Z.N.A. Total RNA Kit I. The RNA quantity was determined using the Nano-Drop ND-1000 spectrophotometer (Wilmington, NC, USA). First strand cDNA synthesis was carried out using the high-capacity cDNA RT kit (Thermo Fisher Scientific, Walthman, MA, USA). Maxima SYBR Green qPCR Master Mix (Thermo Fisher Scientific, Walthman, MA, USA) was used for the real time-RT-PCR, with 250 nM of each primer DEFB1 (HP208395), DEFB4A (HP208178), CAMP (HP207673), Lactoferrin (HP206049), RNASE6 (HP208722), RNASE7 (HP216036) (OriGene, MD, USA) and 5 ng cDNA. The CFX96 Touch Real-Time PCR Detection System (Bio-Rad Laboratories, Hercules, CA, USA) was used for the PCR (initial denaturation at 95 °C for 10 min, 40 cycles of denaturation at 95 °C for 15 s, followed by annealing/extension at 60 °C for 60 s). The Ct (ΔΔCt) method was used to analyze the Ct-valuates, and GAPDH was used as a housekeeping gene. Fold difference was calculated as 2^−ΔΔCt^, as previously described [18].

### 2.9. Western Blot Analysis

The bladder epithelial cells were lysed in radioimmunoprecipitation assay (RIPA) buffer supplemented with a protease and phosphatase inhibitor mix (Thermo Fisher Scientific, Walthman, MA, USA). The protein concentration was assessed with the DC protein assay (Bio-Rad Laboratories, Hercules, CA, USA). Laemmli buffer was mixed with equal amounts of proteins, boiled for 5 min in 95 °C and separated on a 4–20% SDS-polyacrylamide gel electrophoresis. The proteins were then transferred onto a polyvinylidene fluoride membrane (PVDF) (Bio-Rad Laboratories). A quantity of 3% bovine serum albumin in Tris-buffered Saline 0.1% Tween 20 (TBST) was used to block the PVDF membrane for 1 h at room temperature and incubated over night at 4 °C with the primary antibodies. Human estrogen receptor alpha was detected by using a rabbit monoclonal antibody (Thermo Fisher Scientific, Walthman, MA, USA). Human estrogen receptor beta was detected by using a rabbit polyclonal antibody (Thermo Fisher Scientific, Walthman, MA, USA). Human NLRP3 was detected by using a rabbit monoclonal antibody (Cell signaling Technologies, MA, USA). GAPDH was detected by using a rabbit polyclonal antibody (Santa Cruz Biotechnology, Dallas, TX, USA). As secondary antibodies, goat anti-rabbit IgG (HRP) (Santa Cruz Biotechnology) was used and incubated for 1 h at room temperature. The bands were visualized using Luminata Forte Western HRP Substrate (Merck Millipore, Darmstadt, Germany), as previously described [27].

### 2.10. Luciferase Assay

Bladder epithelial cells were transfected with the 3X ERE TATA luc plasmid (a gift from Donald McDonnell, Addgene plasmid #11354) [31] and the renilla luciferase-expressing construct (pGL4.74, Promega, Madison, WI, USA) using lipofectamine 2000. The cells were then stimulated with 1 nM estradiol for 24 h, incubated at 37 °C with 5% CO_2_. Cell lysates were assayed and measured for bioluminescence using the Pierce™ Renilla-Firefly Luciferase Dual Assay Kit in a Lumi-star Omega machine (BMG Labtech Aylesbury, Aylesbury, UK), according to the manufacturer’s instructions. The renilla luciferase-expressing plasmid (pGL4.74) was used to normalize the transfection.

### 2.11. Colonization Assay

Bladder epithelial cells were stimulated with 1 nM or 10 nM estradiol for 24 h at 37 °C with 5% CO_2_. DMSO was used as a negative control. After 24 h, bladder epithelial cells were infected with CFT03 (carrying an enhanced GFP-expressing plasmid, eGFP) at MOI 10 for 4 h at 37 °C and 5% CO_2_. This was carried out to evaluate bacterial colonization (adherent and intracellular bacteria) of 5637 cells. The cells were then washed ten times with PBS, and the fluorescence (eGFP) was measured using the Cytation 3 plate reader. Colonization is presented as mean fluorescence intensity (MFI).

### 2.12. Invasion Assay

Bladder epithelial cells were stimulated with 1 nM or 10 nM estradiol for 24 h at 37 °C with 5% CO_2_. DMSO was used as a negative control. The cells were then infected with CFT073 (MOI 10) for 2 h at 37 °C and 5% CO_2_. The cells were then washed ten times with PBS. To kill the remaining of the extracellular CFT073, DMEM with 100 μg/mL gentamicin was added to the cells for 2 h. The plate was then washed three times and the 5637 cells were lysed with 0.1% Triton-X 100 in PBS (with Calcium chloride 100 mg/L and Magnesium chloride 100 mg/L). Lastly, CFT073 were plated on TSA plates and counted next morning after incubation at 37 °C, as previously described [27].

### 2.13. Data Processing and Statistical Methods

The presented data are shown as mean ± standard error of the mean (SEM). Differences between groups were assessed by unpaired Student’s t-test or one-way ANOVA, followed by Bonferroni multiple testing correction. Results were regard as statistically significant at *p* < 0.05. *n* = number of independent experiments. Microarray analysis was performed using Gene Spring GX v. 14.9 (Agilent Technologies, Palo Alto, CA, USA) after per chip and gene 75th percentile shift normalization of samples. Different expression between groups was analyzed with one-way analysis of variance (ANOVA) parametric test. Significantly expressed entities (*p* < 0.05) were obtained by TukeyHSD post-hoc test, followed by Benjamini-Hochberg multiple testing correction and a fold change set at ≥2. GO enrichment analyses were conducted with Cytoscape 3.9.1 with STRING (v. 11.5) enrichment with a false discovery rate (FDR) of <0.05.

## 3. Results

Gene Expression in CFT073-Infected Bladder Epithelial Cells

**Figure 1:**
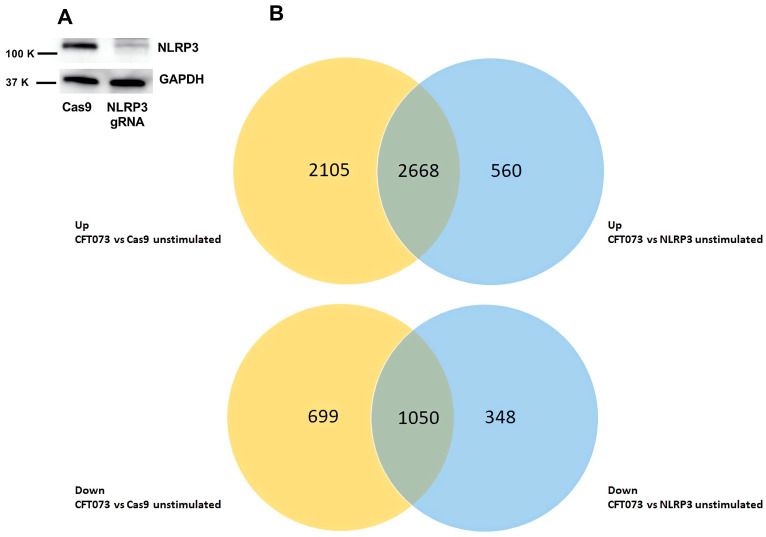
Gene expression alterations after CFT073 infection. Western blot analysis showing deficiency of NLRP3 in bladder epithelial cells using the CRISPR/ Cas9 system (**A**). Cas9 and NLRP3-deficient cells were infected with CFT073 for 6 h, followed by a microarray analysis. Venn diagram is showing the number of differentially expressed gene entities that were upregulated (Up) or downregulated (Down) after CFT073 infection of Cas9 control cells (yellow) or NLRP3-deficient cells (blue) compared to unstimulated cells (**B**).

Bladder epithelial cells deficient in NLRP3 were constructed with the CRISPR/Cas9 system as previously described (Figure 1A) [19,21,27]. A microarray analysis was performed on mRNA isolated from bladder epithelial cells stimulated with CFT073 at MOI 10 for 6 h. The differentially expressed gene entities are visualized using a Venn diagram (Figure 1B). Upon CFT073 infection of Cas9 control cells, 4773 gene entities were upregulated, and 1749 gene entities were downregulated (*p* < 0.05) with at least a ≥2-fold change compared to unstimulated Cas9 cells (Figure 1B). CFT073 infection of NLRP3-deficient cells significantly upregulated 3228 gene entities and downregulated 1398 gene entities compared to unstimulated NLRP3-deficient cells (Figure 1). All significantly altered gene entities are listed in Supplementary Table S1 (upregulated) and Supplementary Table S2 (downregulated).

## 4. Gene Ontology Analysis

**Figure 2:**
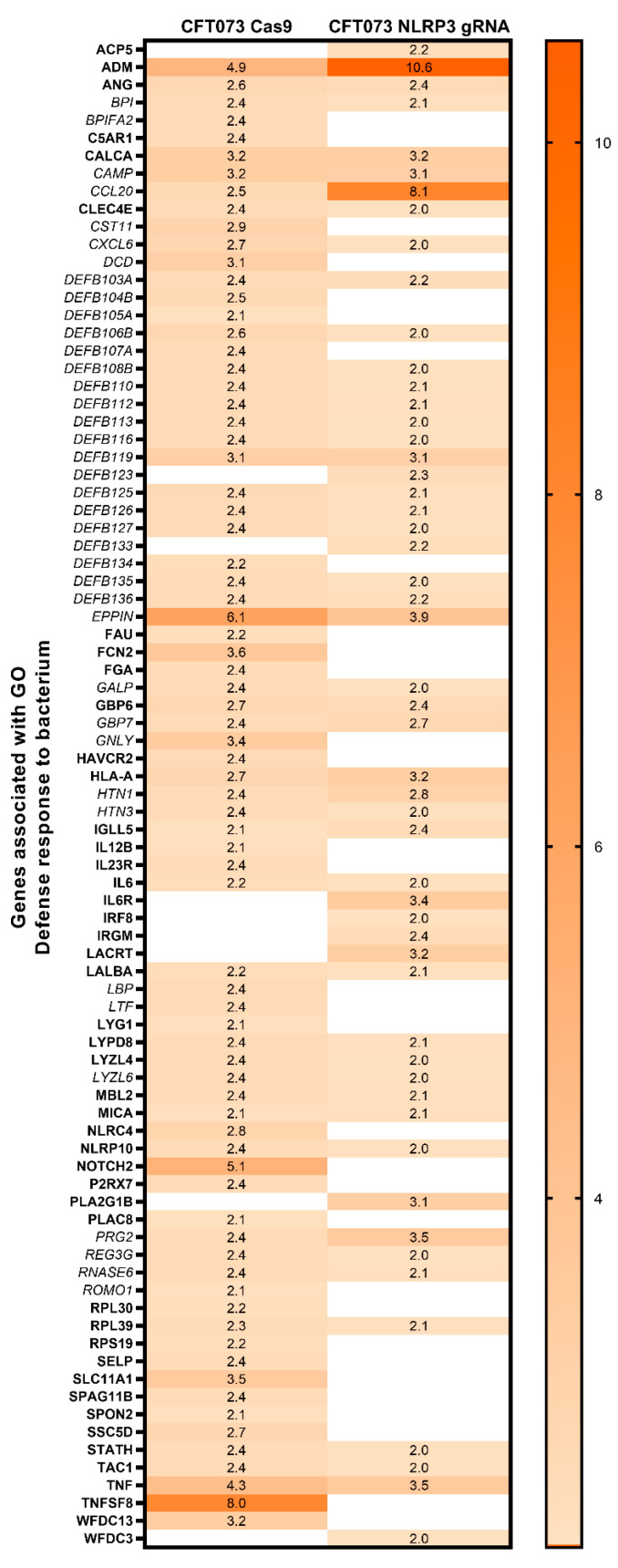
Genes associated with gene ontology Defense response to bacterium. Heat map showing the gene profile of 85 significantly upregulated genes (*p* < 0.05) in CFT073-infected Cas9 or NLRP3-deficient cells (NLRP3 gRNA) after 6 h, compared to unstimulated cells. All proteins and peptides with antimicrobial activity are highlighted in italics.

We continued to evaluate the microarray data with gene ontology analysis. In total, 229 upregulated (Supplementary Table S3) and 252 downregulated (Supplementary Table S4) gene ontologies were significantly enriched. Out of these, the gene ontology Defense response to bacterium was chosen for further evaluation (Figure 2). Out of 85 gene entities, CFT073-infected Cas9 cells significantly increased the expression of 76 gene entities (of which 66 entities were higher expressed in Cas9 compared to NLRP3-deficient cells), and CFT073-infected NLRP3-deficient cells significantly increased the expression of 53 gene entities (of which 16 entities were higher expressed in NLRP3-deficient cells compared to Cas9 cells) (Figure 2).

### 4.1. NLRP3 Affects the Growth of CFT073

**Figure 3:**
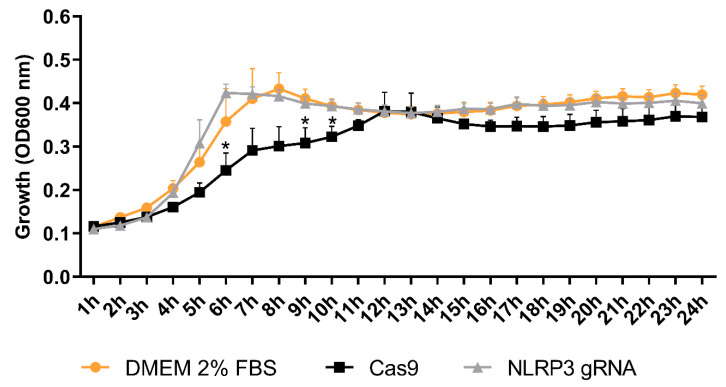
Bacterial growth evaluation. CFT073 was exposed to conditioned medium from Cas9 controls and NLRP3-deficient cells (NLRP3 gRNA), and the bacterial growth was evaluated with optical density at 600 nm. Data are shown as mean ± SEM of three independent experiments. Asterisks show statistical significance (* *p* < 0.05) compared to NLRP3 gRNA.

The gene ontology analysis showed that the expression of 32 out of 39 antimicrobial peptides was reduced in CFT073-infected NLRP3-deficient cells compared to Cas9 cells. We therefore continued to investigate the effect of conditioned medium from NLRP3-deficient cells on the growth of CFT073 for 24 h. Conditioned medium from Cas9 control cells significantly reduced the growth of CFT073 compared to conditioned medium from NLRP3-deficient cells after 6, 9 and 10 h (Figure 3). Conditioned medium from NLRP3-deficient cells did not suppress the growth of CFT073 but instead mimicked the growth pattern of DMEM with 2% FBS (Figure 3).

### 4.2. Involvement of NLRP3 in Antimicrobial Peptide Release

**Figure 4:**
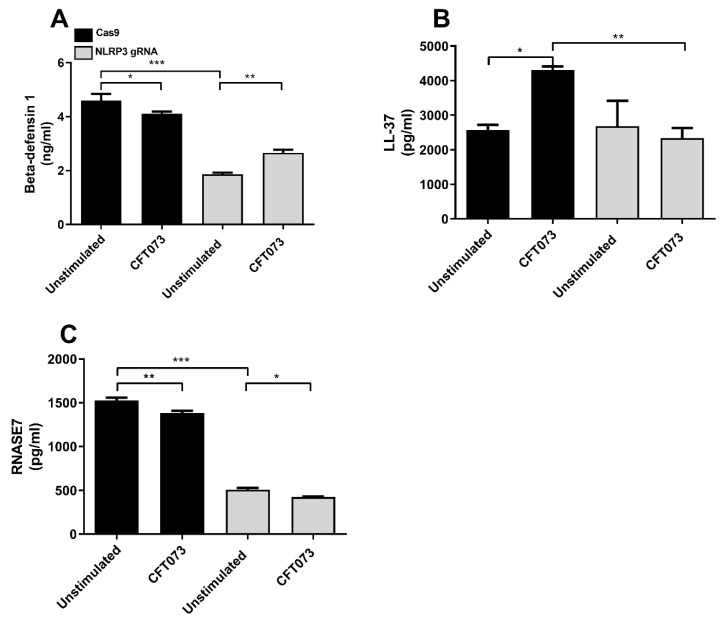
Antimicrobial peptide release after CFT073 infection. Beta-defensin 1 (**A**), LL-37 (**B**) and RNase7 (**C**) release from Cas9 or NLRP3-deficient (NLRP3 gRNA) bladder epithelial cells after stimulation with CFT073 at MOI 10 for 6 h. Data are presented as mean ± SEM of three independent experiments. Asterisks show statistical significance (* *p* < 0.05, ** *p* < 0.01, *** *p* < 0.001).

Next, we investigated the involvement of NLRP3 in the CFT073-evoked release of Beta-defensin 1, Beta-defensin 2, LL-37 and RNase7 from bladder epithelial cells after 6 h of stimulation. CFT073 significantly reduced the release of Beta-defensin 1 (Figure 4A) and RNase7 (Figure 4C) but significantly increased the release of LL-37 (Figure 4B) compared to unstimulated Cas9 cells. The basal release of Beta-defensin 1 and RNase7 was significantly reduced in NLRP3-deficient cells compared to unstimulated Cas9 control cells (Figure 4A,C). In contrast to Cas9 cells, LL-37 was not increased by CFT073 in NLRP3-deficient cells (Figure 4B). NLRP3-deficient cells stimulated with CFT073 were able to induce a significant increase in Beta-defensin 1 release compared to unstimulated NLRP3-deficient cells (Figure 4A). Moreover, CFT073 induced a significantly decreased RNase7 release from both Cas9 controls and NLRP3-deficient cells compared to unstimulated cells (Figure 4C). Release of Beta-defensin 2 was not detected from bladder epithelial cells after CFT073 infection for 6 h.

### 4.3. Involvement of NLRP3 in Estradiol-Induced Expression and Release of Antimicrobial Peptides

**Figure 5:**
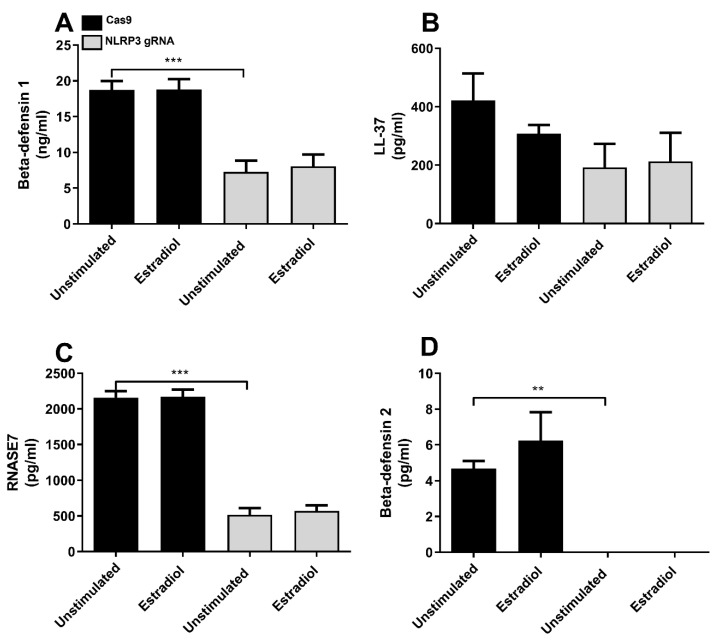
The effect of estradiol on antimicrobial peptide release. Beta-defensin 1 (**A**), LL-37 (**B**), RNase7 (**C**) and Beta-defensin 2 (**D**) release from Cas9 or NLRP3-deficient (NLRP3 gRNA) bladder epithelial cells stimulated with estradiol (10 nM) for 24 h. Data are presented as mean ± SEM of four independent experiments. Asterisks show statistical significance compared to respective unstimulated control (** *p* < 0.01, *** *p* < 0.001).

Estradiol has been linked to the release of AMPs from the urinary tract [32,33] and given the effect of NLRP3 on AMP release, we wanted to investigate if NLRP3 could be involved in regulating the estradiol-mediated expression and release of AMP from bladder epithelial cells. Bladder epithelial cells were stimulated with 10 nM estradiol for 24 h and the release of Beta-defensin 1, LL-37, RNase7 and Beta-defensin 2 were measured. Estradiol did not increase the release of AMPs from Cas9 cells or NLRP3-deficient cells at the protein level after 24 h (Figure 5). However, in line with our previous results, NLRP3 affected the basal release of Beta-defensin 1 (Figure 5A), RNase7 (Figure 5C) and Beta-defensin 2 (Figure 5D).

**Figure 6:**
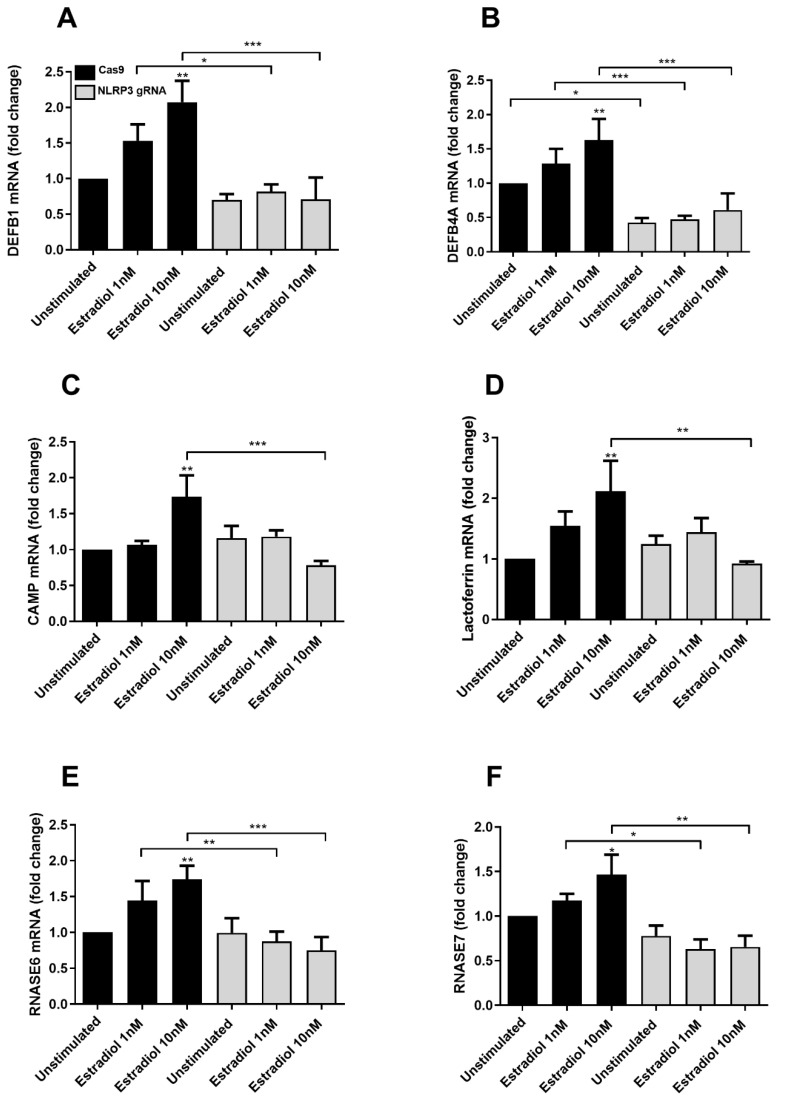
Gene expression of antimicrobial peptides. Cas9 and NLRP3-deficient (NLRP3 gRNA) bladder epithelial cells were stimulated with 1 nM or 10 nM estradiol for 24 h, followed by analysis of DEFB1 (**A**), DEFB4A (**B**), CAMP (**C**), Lactoferrin (**D**), RNASE6 (**E**), RNASE7 (**F**) mRNA expression. The gene expression was normalized to GAPDH and expressed as fold change relative to unstimulated Cas9 controls. Data are presented as mean ± SEM of three independent experiments. Asterisks show statistical significance compared to unstimulated control (* *p* < 0.05, ** *p* < 0.01, *** *p* < 0.001).

Next, we continued to evaluate the expression of different AMPs at the gene level after estradiol stimulation. Bladder epithelial cells were stimulated with 1 nM or 10 nM estradiol for 24 h, and the mRNA expression of DEFB1 (Beta-defensin 1), DEFB4A (Beta-defensin 4A), CAMP (LL-37), Lactoferrin, RNASE6 and RNASE7 was analyzed. Estradiol significantly increased the gene expression of DEFB1, CAMP, Lactoferrin, RNASE6, RNASE7 and DEFB4A in Cas9 control cells (Figure 6). However, the estradiol-induced increase of AMPs was abrogated in NLRP3-deficient cells (Figure 6). We also found that DEFB4A, but not DEFB1, RNASE6, RNASE7 or CAMP, had significantly lower basal gene expression in the NLRP3-deficient cells compared to Cas9 control cells.

### 4.4. Expression of Estrogen Receptors in NLRP3-Deficient Cells

**Figure 7:**
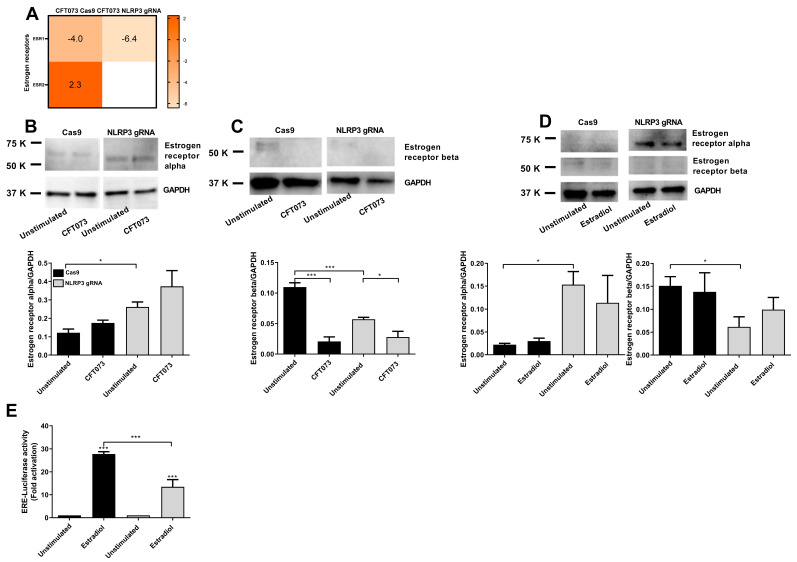
Expression and activity of estrogen receptors. Heat map showing the gene profile of ESR1 and ESR2 (*p* < 0.05) in CFT073–infected Cas9 or NLRP3–deficient cells (NLRP3 gRNA) after 6 h compared to unstimulated cells (**A**). Cas9 and NLRP3-deficient bladder epithelial cells were stimulated with CFT073 at MOI 10 for 6 h (**B**,**C**) or 10 nM estradiol for 24 h (**D**), followed by western blot analysis. GAPDH was used as a loading control. Estrogen receptor activity after stimulation with 1 nM estradiol for 24 h was evaluated with the ERE-luciferase reporter assay (**E**). Data are presented as mean ± SEM of three independent experiments. Asterisks show statistical significance compared to unstimulated cells (* *p* < 0.05, *** *p* < 0.001).

We proceeded with evaluating the expression of estradiol receptors in NLRP3-deficient cells in order to understand the mechanism behind the reduced AMP expression. The microarray data showed that CFT073 significantly decreased the expression of estrogen receptor 1 (ESR1) in both Cas9 control cells and NLRP3-deficient cells compared to unstimulated cells after 6 h (Figure 7A). CFT073 increased the expression of estrogen receptor 2 (ESR2) in Cas9 control cells but not in NLRP3-deficient cells (Figure 7A). Furthermore, the basal protein expression of estrogen receptor alpha (ESR1) was significantly increased, and the basal expression of estradiol receptor beta (ESR2) was significantly decreased in NLRP3-deficient cells compared to Cas9 cells (Figure 7B,C). The estradiol receptor beta was significantly downregulated by CFT073 in both Cas9 cells and NLRP3-deficient cells (Figure 7C). We then continued to evaluate the protein expression of estrogen receptors alpha and beta after stimulation with estradiol for 24 h (Figure 7D). Estradiol did not change the protein expression of estrogen receptor alpha or beta compared to unstimulated cells in Cas9 or NLRP3-deficient cells (Figure 7D). However, the basal expression of the alpha receptor was significantly increased, and the basal expression of the beta receptor was significantly decreased in NLRP3-deficient cells compared to Cas9 cells (Figure 7D). As the protein expression of the two receptor subtypes was oppositely altered, further evaluation to reveal the overall response to estradiol was needed. An ERE-luciferase reporter assay was used to evaluate the estrogen receptor activity after estradiol stimulation. This assay showed that the estrogen receptor activity was significantly lower in NLRP3-deficient cells compared to Cas9 cells after estradiol stimulation for 24 h (Figure 7E).

### 4.5. The Role of Estradiol in Bacterial Invasion of Bladder Epithelial Cells

**Figure 8:**
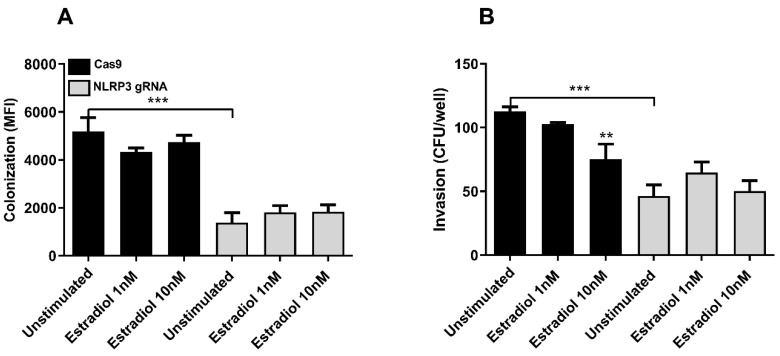
Bacterial colonization and invasion of bladder epithelial cells. Cas9 and NLRP3-deficient (NLRP3 gRNA) bladder epithelial cells were stimulated with 1 nM or 10 nM estradiol for 24 h, followed by stimulation with CFT073, and bacterial colonization and invasion were evaluated. Bacterial colonization with CFT073 (harboring a GFP-expressing plasmid) was quantified as mean fluorescence intensity (MFI) (**A**). Bacterial invasion was measured using the gentamycin protection assay and the invasion quantified as the number of intracellular bacteria (CFU/well) (**B**). Data are shown as mean ± SEM (*n* = three independent experiments). Asterisks show statistical significance compared to unstimulated cells (** *p* < 0.01, *** *p* < 0.001).

Estradiol is known to have a protective effect against the formation of intracellular bacterial reservoirs. Estradiol promotes bladder epithelial integrity and supports the production of antimicrobial peptides [32]. We next evaluated if NLRP3 could be involved in the estradiol-mediated protection of bladder epithelial cells against CFT073 colonization. Bladder epithelial cells were pre-treated with 1 nM and 10 nM estradiol for 24 h, followed by CFT073 infection. Pre-treatment with estradiol did not significantly protect the bladder epithelial cells from colonization (Figure 8A). However, bacterial colonization of NLRP3-deficient cells was significantly lower than colonization of Cas9 cells (Figure 8A). Furthermore, estradiol pre-treatment significantly reduced bacterial invasion of Cas9 cells but not of NLRP3-deficient cells (Figure 8B). In addition, unstimulated NLRP3-deficient cells had less intracellular CFT073 compared to unstimulated Cas9 cells (Figure 8B).

## 5. Discussion

The NLRP3 inflammasome has been emphasized to be an important modulator of the early host defense in the urinary tract against UPEC [13,15,19]. In this study, we showed that cells lacking NLRP3 had a differential expression of several gene ontologies, mainly associated with immune response activity, defense response to bacterium, cell–cell signaling and cytokine and hormone activity. Thus, NLRP3 seems to have an extensive role in regulating gene expression in bladder epithelial cells that is not only restricted to the classical regulation of pro-inflammatory cytokines. This is in agreement with our previous findings showing that NLRP3 regulates cytokine and chemokine release from bladder epithelial cells and also affects the antimicrobial activity of neutrophils [21]. During the early phase of UPEC infection, the host cells release different factors, such as cytokines and chemokines (IL-1β, IL-6, IL-8 and TNF-α) and hormones (norepinephrine, estradiol), to fight off the infection [34,35]. However, UPEC may suppress NLRP3 in bladder epithelial cells by the virulence factor TcpC and thus produce a compromised NLRP3-deficent bladder milieu resembling our experimental cell model [20]. Based on this, the gene ontology Defense response to bacterium was chosen for further evaluation. Most of the genes in this gene ontology showed higher expression in Cas9 cells compared to NLRP3-deficent cells after CFT073 infection. Several of the genes with low expression in the NLRP3-deficient cells belonged to antimicrobial peptides such as defensins, CAMP and RNASE6. Antimicrobial peptides, such as LL-37, have previously been linked to NLRP3 inflammasome activation [36].

The release of antimicrobial peptides is an important part of the body’s first line of defense against UPEC [37]. They have powerful immunomodulatory effects, including the induction of cytokines, chemokines and tight junction proteins [38,39]. Beta-defensins are expressed in the urogenital tract; however, their role during a UTI is not completely understood [22]. RNase7 has broad-spectrum antimicrobial activity against uropathogenic bacteria, and its expression increases during an infection [40]. LL-37 is increased among patients with UTIs [41], and it is known to suppress UPEC growth and protect against UPEC adherence in the urinary tract [42]. NLRP3 was found to be involved in the basal release of Beta-defensin 1 and RNase7, but not LL-37, from bladder epithelial cells. NLRP3 was also found to be essential for CFT073-induced increased LL-37 release from bladder epithelial cells. In order to understand if the reduced expression of antimicrobial peptides functionally affects bacterial growth, conditioned medium from Cas9 and NLRP3-deficient cells was collected and the growth of CFT073 was evaluated. The result showed that conditioned medium from Cas9 cells suppressed the growth of CFT073, but that conditioned medium from NLRP3-deficient cells failed to suppress growth. This indicates that NLRP3 is important for suppressing UPEC growth. Our results indicate that NLRP3 is involved in regulating the basal expression and release of AMPs in bladder epithelial cells, which, to our knowledge, has not been shown previously. The severity of a UTI caused by clinical E. coli isolates was observed to be higher in cases where the isolates were more resistant to LL-37. This indicates that LL-37 is a crucial component of mucosal immunity in the urinary tract [42]. The NLRP3-deficent bladder milieu mediated by the UPEC virulence factor TcpC might hence lead to more severe UTIs due to decreased AMP levels.

Estradiol has been linked to both the release of AMPs from the urinary tract [22,23] and to the regulation of NLRP3 activation [43,44,45]. Since estradiol, AMPs and NLRP3 are interlinked, it was of interest to evaluate if NLRP3 could be involved in regulating estradiol-mediated AMP expression from bladder epithelial cells. Estradiol did not induce an increased release of Beta-defensin 1, LL-37, RNase7 or Beta-defensin 2 from bladder epithelial cells at the protein level. However, the mRNA expression of Beta-defensin 1, Beta-defensin 4A, CAMP, Lactoferrin, RNASE6 and RNASE7 was increased by estradiol. Interestingly, the estradiol-induced increase in mRNA expression was not noted in NLRP3-deficient cells. Han et al. showed that Beta-defensin 1 is produced in a constitutive manner in vaginal epithelial cells and that estradiol did not influence the production of Beta-defensin 1 or Beta-defensin 2 during normal conditions. However, estradiol in combination with LPS could induce the production of Beta-defensin 2 in vaginal epithelial cells [33]. Hence, the upregulation of AMP from bladder epithelial cells may need a synergistic signaling from both estradiol and UPEC (LPS) in order to induce increased peptide release. Taken together, our data suggests an involvement of NLRP3 in estradiol-mediated AMP gene expression, which has not previously been shown.

Estradiol can bind to two types of nuclear estrogen receptors, ER alpha and ER beta, and these receptors have different functions and tissue expression patterns [24,25]. We hypothesized that NLRP3 may affect the expression of these receptors and thereby affect estradiol-induced AMP expression. The basal protein expression of ER alpha was significantly increased, whereas ER beta was significantly decreased in NLRP3-deficient cells in contrast to Cas9 cells. We also showed that ER beta was significantly downregulated by CFT073 in both Cas9 cells and NLRP3-deficient cells. However, estradiol did not alter the protein expression of ER alpha or beta in Cas9 or NLRP3-deficient cells. We proceeded to evaluate the involvement of the alpha and beta receptors using a ERE-luciferase reporter assay to clarify the activity outcome of the oppositely altered receptor expressions. These experiments showed that the estrogen receptor activity, induced by estradiol, was significantly lower in NLRP3-deficient cells compared to Cas9 cells. Hence, our results suggest that ER beta, which is reduced in NLRP3-deficient cells, is most likely the receptor subtype by which NLRP3 regulates estradiol-mediated AMP expression. It has been shown that ER beta is the major ER type in the urinary bladder [24,25], and that ER alpha is expressed preferentially in the vagina [25,26]. Hence, we and others have shown that UPEC can directly or indirectly, through TcpC downregulation of NLRP3 [20], inhibit UPEC/estradiol-induced AMP release from bladder epithelial cells. This indicates that UPEC has developed several evasion mechanisms to suppress AMP release to successfully colonize the bladder. Our findings also highlight that an NLRP3-deficent bladder milieu resembles a local state of menopause in the bladder. Although estrogen is present, the protective effect of estrogen is reduced due to the downregulation of the ER beta.

UPEC ability to colonize and remain in the bladder depends on a complex interplay between the bacteria and host responses, such as estradiol. UPEC has the ability to injure the superficial epithelial cell layer, which exposes the underlying cell layer to bacteria. This provides an opportunity to form persistent UPEC reservoirs deeper in the urothelium, which is associated with recurrent UTIs [46]. We found that estradiol protects bladder epithelial cells from CFT073 invasion. This protective effect was not observed in NLRP3-deficient cells. This is in agreement with others, who have shown that estradiol strengthens the urothelial integrity [23]. These findings are probably related to the reduced ER beta expression and ER activity as a consequence of NLRP3-deficiency. These findings show that NLRP3 may be important for estradiol-mediated protection against UPEC invasion of bladder epithelial cells.

The causal mechanisms behind our findings are currently unknown, but they may be mediated by primary or secondary effects. NLRP3 has been shown to be a transcriptional regulator of the innate immune response by facilitating the binding of IRF-4 to DNA [47]. Furthermore, the transcription functions of the nucleotide-binding oligomerization domain-like receptor family were described earlier, such as NLRP5 [48] and the major histocompatibility complex class II transactivator CIITA [49]. Furthermore, NLRP3 was found to function as a crucial transcription factor for IL-33 in epithelium independently of inflammasome activation [49]. They found that NLRP3 was primarily located in the nucleus of the epithelial cells in contrast to macrophages, where NLRP3 was found primarily in the cytoplasm [49], supporting a role as transcriptional regulator in epithelial cells.

## 6. Conclusions

In conclusion, our results showed that NLRP3 has a broad role in regulating the host-responses during a UPEC infection in bladder epithelial cells. A novel function of NLRP3 as a possible regulator of estrogen signaling and AMP gene expression was elucidated. Hence, our findings strengthen the notion of NLRP3 as a key player in the defense of the bladder against UPEC. With this knowledge, we understand the host–pathogen interaction better, which may facilitate the development of new strategies to prevent and treat this highly common infection.

## Data Availability

Gene expression data is available in the GEO database with the accession number GSE243098.

## Supplementary Materials

The following supporting information can be downloaded at: https://www.mdpi.com/article/10.3390/cells12182298/s1, Table S1: upregulated genes; Table S2: downregulated genes; Table S3: upregulated gene ontologies; Table S4: downregulated gene ontologies.
